# TCMSID: a simplified integrated database for drug discovery from traditional chinese medicine

**DOI:** 10.1186/s13321-022-00670-z

**Published:** 2022-12-31

**Authors:** Liu-Xia Zhang, Jie Dong, Hui Wei, Shao-Hua Shi, Ai-Ping Lu, Gui-Ming Deng, Dong-Sheng Cao

**Affiliations:** 1 The First Hospital of Hunan University of Chinese Medicine, Changsha, 410007 Hunan People’s Republic of China; 2 Xiangya School of Pharmaceutical Sciences, Central South University, Changsha, 410013 Hunan People’s Republic of China; 3 Advancing Translational Medicine in Bone and Joint Diseases, School of Chinese Medicine, Hong Kong Baptist University, Hong Kong SAR, People’s Republic of China

**Keywords:** Ingredient database, Chinese medicine, Key ingredients, Multi-tool target prediction

## Abstract

Traditional Chinese Medicine (TCM) has been widely used in the treatment of various diseases for millennia. In the modernization process of TCM, TCM ingredient databases are playing more and more important roles. However, most of the existing TCM ingredient databases do not provide simplification function for extracting key ingredients in each herb or formula, which hinders the research on the mechanism of actions of the ingredients in TCM databases. The lack of quality control and standardization of the data in most of these existing databases is also a prominent disadvantage. Therefore, we developed a Traditional Chinese Medicine Simplified Integrated Database (TCMSID) with high storage, high quality and standardization. The database includes 499 herbs registered in the Chinese pharmacopeia with 20,015 ingredients, 3270 targets as well as corresponding detailed information. TCMSID is not only a database of herbal ingredients, but also a TCM simplification platform. Key ingredients from TCM herbs are available to be screened out and regarded as representatives to explore the mechanism of TCM herbs by implementing multi-tool target prediction and multilevel network construction. TCMSID provides abundant data sources and analysis platforms for TCM simplification and drug discovery, which is expected to promote modernization and internationalization of TCM and enhance its international status in the future. TCMSID is freely available at https://tcm.scbdd.com.

## Introduction

Traditional Chinese Medicine (TCM) has played a vital role in extensively treating various diseases for thousands of years in China. In virtue of its exact curative effect, TCM is still used to maintain human health until today and has received worldwide attention. Virtually, TCM restores the human body to normal physiological condition by perturbing the human dysfunctional network through its ingredients, which is consistent with the role of western medicine, despite having a fundamental theory that is very different from that of contemporary western medicine. For example, ephedrine and pseudoephedrine, etc., are regarded as the major ingredients of Ephedra Decoction to treat colds and relieve cough and asthma. Moreover, given the characteristics of multi-ingredient and multi-target, TCM is actually more in line with the trend of current combination therapy of multi-drugs which holds great potential for treating various intractable diseases, such as malignant tumors and cardiovascular diseases rather than the prior concept of ‘one drug-one gene-one disease’ [1].

Herbal ingredients, as the footstone of the TCM, have long been recognized as an ideal starting point for molecular design owing to their broad chemical structural diversity and high selectivity [2]. It is estimated that no less than 50% approved small-molecular clinical drugs in the world are directly or indirectly derived from herbal ingredients [3, 4]. Artemisinin extracted from *Artemisia annua* is a well-known example to treat malaria. However, it is the multi-ingredient and multi-target characteristics of the TCM that make the mechanism of action remain exclusive, thus hindering its application and modernization [5, 6].

The common strategy for TCM mechanism exploration is to map all the ingredients of a prescription to the ingredient-target network and infer probable mechanisms of the prescription from enriched pathways [7–9]. It is well known that there are tens of thousands of ingredients in TCM, even the whole chemical information of an herb/prescription from TCM itself can be treated as a database of natural products; however, most of the ingredients are ineffective and redundant and rarely or even do not exert effective pharmacological responses owing to their low content and activity. Moreover, the reliability of the target is critical to revealing the mechanism. The rough strategy used above only depicts an impractical blueprint instead of deciphering the real mechanisms of TCM, which makes the inherently complex mechanism more confusing and hinders the study of mechanism. Therefore, the simplification step for TCM ingredients is imperative for clarifying mechanisms and promoting the development of TCM.

Over the past decade, with the emergence of several herbal ingredient databases, such as TCM@Taiwan [10], TCMSP [11], TCMID [12], etc., acquisition of TCM ingredient information has no longer been limited to articles, which have largely facilitated the subsequent mechanism study and TCM modernization process. However, these databases inevitably have several certain shortcomings. Despite the fact that TCM@Taiwan increased the number of his component records to 6100 in 2014, it still lacks TCM classification and compound-target data. HIT is a target-focused database with limited ingredients data. TCMSP is a comprehensive database containing a high number of records for TCM ingredients, targets, diseases, and even ADME, however, it does not contain TCM classification and quality control methods. The number of records for each item of TCMID is even greater than that of TCMSP and TCMSID. Even though it collected MS data of 3895 TCM ingredients to conduct quality control, TCMID 2.0 did no herb classification. ETCM is another TCM database with comprehensive data types, which included herb classification, ingredient data, target data, disease data, quality control, network display and even TCM pictures. However, the number of entries of nearly every data type is less than TCMSP, TCMID 2.0 and TCMSID respectively. NPASS and NPACT are characterized by the experimental data load they included, yet the two databases included no TCM classification, ADME/T information and quality control method. While each of these databases has its own advantages and complements each other, TCMSID includes almost all of the advantages. More importantly, TCMSID provided simplification function for extracting key ingredients in each herb or formula, which is the unique and significant trait of the database [13]. Although some databases provide mechanism analysis function, most of them reveal the pharmacological mechanism of the whole ingredients of an herb/prescription based on a rough mapping strategy, which only results in ambiguous mechanism. Present-existing major databases of TCM ingredients/natural products and their basic characteristics are shown in Table 1.

**Table 1:** Present-existing major databases of TCM ingredients/natural products and their basic characteristics

Database name	TCM	Ingredients	Target	ADME/T	Experimental bioactivity data	Quality control	Website
TCM@Taiwan	453	> 20,000	N/A	N/A	N/A	N/A	http://tcm.cmu.edu.tw
HIT	1300	586	1301	N/A	N/A	N/A	http://lifecenter.sgst.cn/hit/
TCMSP	499	29,384	3311	Yes	N/A	N/A	http://tcmspw.com/news.php
TCMID2.0	8159	43,413	17,521	N/A	N/A	Less	http://www.megabionet.org/tcmid/
ETCM	403	7274	7603	Yes	N/A	Yes	http://www.nrc.ac.cn:9090/ETCM/
NPASS	N/A	35,032	5863	N/A	Yes	N/A	http://bidd2.nus.edu.sg/NPASS/
NPACT	N/A	1574	284	Yes	Yes	N/A	http://crdd.osdd.net/raghava/npact/

**Fig. 1:**
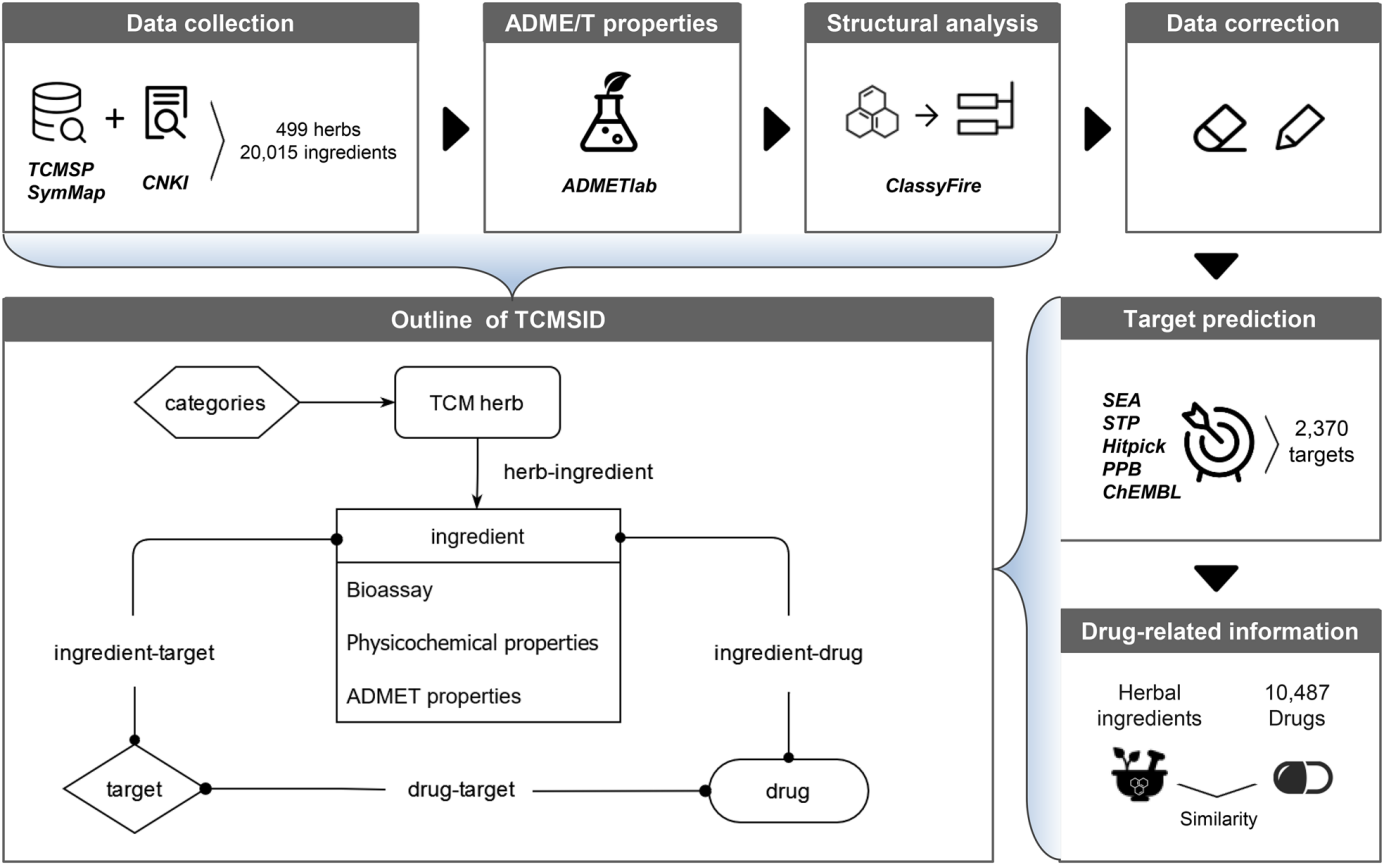
The construction workflow of TCMSID.

In this study, we developed TCMSID, a Traditional Chinese Medicine Simplified Integrated Database (https://tcm.scbdd.com). TCMSID is a database of high storage, high quality and standard, which is specifically manifested in the following aspects: (1) integration of 499 TCM herbs and 20,015 unique herbal ingredients, which largely compensates for the prior-existing databases; (2) the adjunction with multiple aspects of comprehensive information for each ingredient contained in TCM herbs, including significance degree, ADME/T-related properties, structural classification and reliability; (3) the incorporation of reliable potential targets predicted by multiple target-prediction platforms for each ingredient; (4) the provision of bioassay data for herbal ingredients, which can be used to study hidden activity-related information using cheminformatics methods; (5) the establishment of an herb-component-target-drug multilevel interaction network of TCM for deeper study of the mechanism of actions. A summary of the construction workflow of TCMSID is shown in Fig. 1.

Most importantly, TCMSID is not only a repository of TCM ingredients available for query purpose, but also an analysis platform to facilitate clarifying the mechanism of actions. Key ingredients can be screened out as representative ingredients for pharmacological activities exerted by respective TCM herbs and used for multi-tool target prediction to obtain reliable targets to finally clarify the whole mechanism. Also, it provides data analysis and visualization of TCM related information on the network level. The data volume of TCMSID is summarized in Table 2.

**Table 2:** Data source and volume of TCMSID

Item	Data source	Amount of data
TCM herbs	TCMSP, SymMap	499
Total ingredients	Literature mining, TCMSP, SymMap	20,015
Herb-ingredient associations	Literature mining, TCMSP, SymMap	50,053
Focused targets	SEA, Swiss targetprediction, HitPickV2, PPB, PPB2, ChEMBL	2390
Drugs	DrugBank	10,487

## Implementation and functionalities

TCMSID is composed of five fields, including TCM categories, TCM herb, ingredient, target and drug (Fig. 1). Detailed information of each field was integrated from other relevant databases, text mining of published articles and prediction tools such as ADMETlab [14, 15]. In virtue of these interrelated fields, users can conduct a query relying on keywords of any field as an entry point and retrieve relevant information as needed based on the corresponding links. To conduct TCM simplification and mechanism analysis, representative key ingredients of an herb, which exert the pharmacological action of the herb, are available to be screened out. The identification method is based on the detailed information about the ingredients, mainly including significance degree, ADME/T, physicochemical properties, structural reliability, and structural characteristics. Meanwhile, a multilevel functional network can be built through the resulting key ingredients, the reliable targets of the key ingredients and the similar-drug-related information of the key ingredients. This network bridges the gap between TCM and modern medicine. Next, we will elaborate on the detailed information and acquisition process for constructing TCMSID concluded in Fig. 1.

### Data processing and implementation

#### Herbal ingredients

To ensure the high storage of the database, 499 frequently used and approved TCM herbs were collected from the Pharmacopoeia of the People’s Republic of China (2015 version). It is well known that a TCM herb is more likely contains hundreds of compounds and can even be regarded as a small compound library, however, not all the ingredients contained are pharmacologically active. Herein, to extract the major active ingredients of TCM herbs, more than 1500 Chinese articles researching these TCM herbs were retrieved from China National Knowledge Infrastructure (CNKI) (http://www.cnki.net/), since TCM was widely used and researched in China and the related research results were mainly published in Chinese as well. The ingredients with high content and activity were extracted through manual mining of these literatures. The herbal ingredients from those publications, as well as from other related web-based databases including TCMSP and SymMap [16] form the data foundation of TCMSID. The significance degree ranges from 0 to 2, the smaller the number, the higher the significance degree. The three numbers are assigned by the bioactivity data and the minimum volume of a compound per unit to exert pharmacological effect according to the referred literature and the Pharmacopoeia of the People’s Republic of China (2015 version), respectively. For the compound that satisfies the criteria of both bioactivity and minimum volume per unit, we assign the degree of significance the value of 0; for the compound that satisfies the criteria of either bioactivity or minimum volume per unit, we assign the degree of significance the value of 1; and for the compound that fails to satisfy the criteria of both bioactivity and minimum volume per unit, we assign the degree of significance the value of 2. The data of bioactivity and minimum volume per unit for the 499 TCM herbs was manually collected from the Pharmacopoeia and literature, following which the significance degree values of all the ingredients were assigned according to the aforementioned criteria. Details about those ingredients, such as name, structure etc., were comprehensively retrieved from PubChem (PUG-REST interface) automatically [17, 18], where the structure files in multiple formats (sdf, mol, SMILES etc.) were eventually converted into canonical SMILES using OpenBabel (version 2.4.1). The duplicates were removed according to InChIKey.

#### ADME/T-related properties

To improve the quality of the database, we conducted an in-depth analysis for each ingredient. First of all, a battery of pivotal drug-likeness properties were computed through our prior work ADMETlab (http://admet.scbdd.com) and ADMETlab 2.0 (https://admetmesh.scbdd.com/), including ADME/T parameters: Caco-2 permeability (Caco-2), Bioavailability (F-30), Plasma Protein Binding (PPB), Blood-Brain Barrier (BBB) Penetration, Half Life (T_1/2_), Clearance (CL), hERG Inhibition (hERG), Human Hepatotoxicity (HHT), drug-likeness (DL), etc. and basic physicochemical parameters: molecular weight (MW), LogP, LogS, etc. Different from most of the property computational tools, ADMETlab and its updated version is an ADME/T evaluation platform, which integrates comprehensive ADME/T properties and basic physicochemical endpoints as many as possible to provide an overall understanding of query compounds and facilitate the drug discovery process.

Before compounds are further investigated in vitro, ADME/T-related properties and basic physicochemical properties are commonly used to provide a fast preliminary filtering. ADME/T-related properties determine whether a molecule will reach the acting site in the body, and how long it will stay in the bloodstream, while basic physicochemical properties closely related to drug-likeness. Property evaluation is nowadays routinely carried out at the early stage of drug discovery to reduce the attrition rate [19, 20], among which the evaluation of pharmacokinetic and physicochemical properties are important prerequisites for filtering key ingredients. As a result, only the major active ingredients that exhibit favorable pharmacokinetic and physicochemical properties can exert potential biological effects.

#### Ingredient structural classification

To improve the quality of the database, structures of all ingredients were further dissected since the structural characteristic of immense structural diversity is the source of a wide variety of biological activities and the fundamental basis of herbal ingredients for drug design. Herein, ClassyFire web server [21], an automated chemical classification web tool, was used to the refine structural classification of all ingredients layer-by-layer. For instance, matrine, an alkaloid found in plants and a key active ingredient in the herb *Sophora flavescent*, was grouped under the headings of alkaloids and derivatives, lupin alkaloids, and matrine alkaloids.

#### Ingredient structural reliability evaluation

**Fig. 2:**
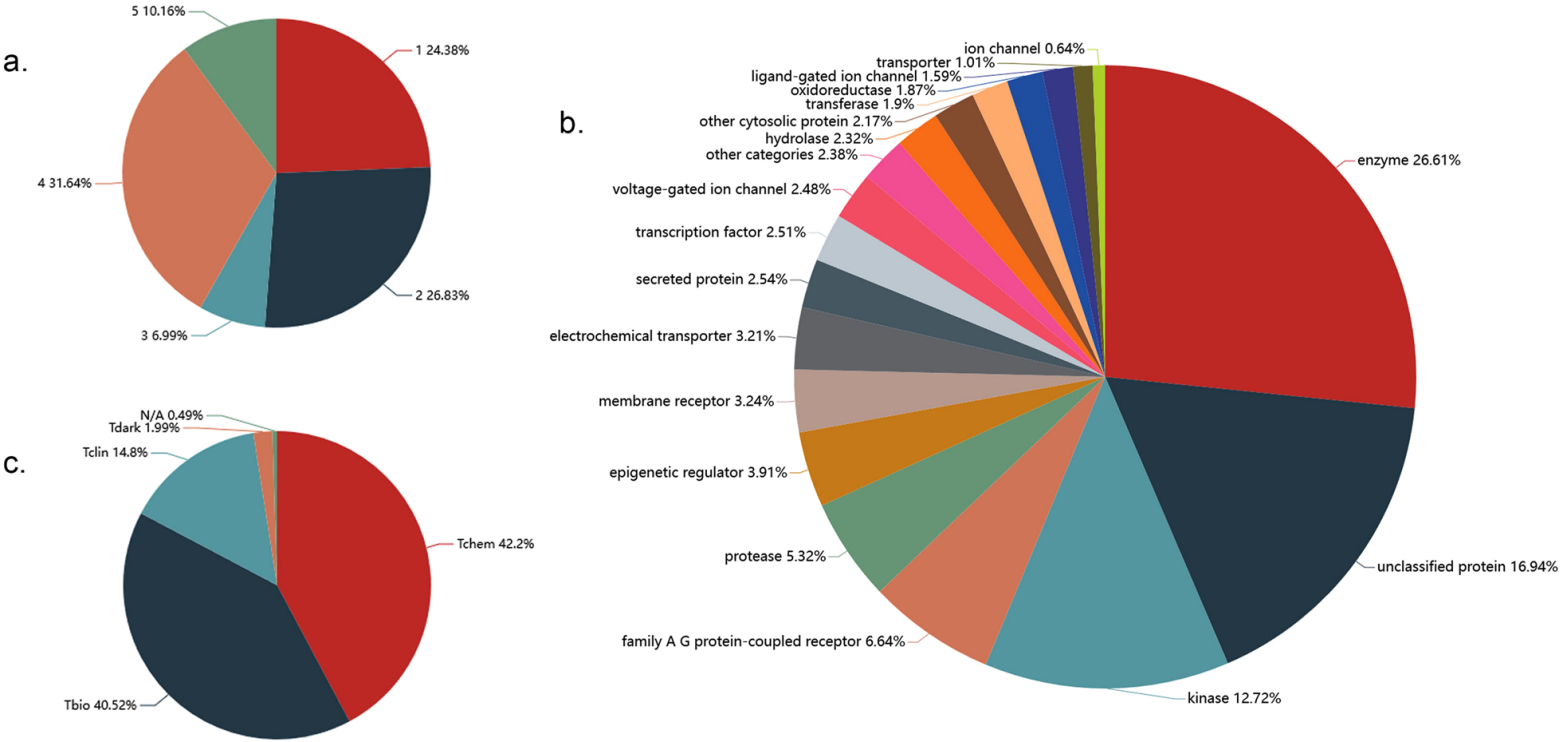
Quality information of TCMSID ingredients shown in (**a**) the structural reliability evaluation of TCM ingredients. And predicted target information shown in (**b**) the classification of ingredient targets by different homologous families and (**c**) the classification of ingredient targets by development levels

From the perspective of structural quality, the structural reliability of ingredients can be trustworthy insufficiently due to the diverse data sources, which will fundamentally hinder the TCM research process in a great measure. To evaluate the structural reliability of each ingredient for accurate analysis, the reliability annotations, which indicate the structural quality, were gained by performing structural reliability evaluation using a semi-automated quality checking workflow while keeping the ingredients failed to meet criteria with structural reliability marking [22]. The operation principle of the workflow is to input the chemical name and CAS number of any Chinese medicine ingredient, and then retrieve data from several different ingredient databases such as PubChem and evaluate the quality of the ingredient data by comparing the consistency of the search results obtained by the two searching methods. Here, the structural reliability ranges from 1 to 5, in which 1 to 3 means relatively higher structural reliability with 1 the highest reliability, while 4 stands for unknown reliability, and only 5 means poor reliability. For the chemicals with unknown reliability, we performed additional manual inspection and information correction, and then rescored the corrected chemicals following the workflow. (Fig. 2a).

#### Ingredient target information

To acquire reliable targets for mechanism exploration, target prediction was performed by implementing and assembling different target prediction tools including SEA [23], SwissTargetPrediction [24], HitpickV2 [25], PPB [26], PPB2 [27] and ChEMBL [28]. We introduced occurrence frequency parameter, which refers to the frequency of targets predicted by different tools. For a given target, the higher the occurrence frequency represents the higher-ranking level. Herein, for each prediction tool, only the top 15 predicted targets were retained according to the occurrence frequency parameter.

Comprehensive information for 3270 target proteins was collected from ChEMBL [29]. Detailed annotation information of all targets is obtained by ID conversion through UniProt [30], which included identification names, functionality description, cross-ref IDs, etc. In addition, target proteins involved in this database were classified into different homologous families through ChEMBL target annotation, such as enzyme and ion channel (Fig. 2b). In the meantime, from a clinical, chemical and biological standpoint, the development level of these targets was divided into Tclin (clinic), Tchem (chemistry), Tbio (biology) and Tdark (dark genome) using TDL classification scheme developed by Oprea et al. (Fig. 2c) [31].

#### Drug-related information

To further clarify the knowledge of TCM functions from the modern medicinal point of view, we built the relationship between TCM ingredients and drugs through chemical similarity. The drugs in TCMSID were collected from the DrugBank database [32], which included a total of 10,450 known drugs (containing 3883 FDA-approved drugs), as well as drug-related information including drug names, structures, and drug targets, etc.

Herein, both FCFP6 and ECFP4 fingerprints were adopted to represent all ingredients and drugs since it was previously reported that the circular fingerprint, especially the FCFP6 and ECFP4, show better performance in TCM ingredient similarity search [33, 34]. As a common measure method for 2D similarity, Tanimoto coefficient (*Tc*) was applied to define chemical structural similarity between comparative individuals. Moreover, *Tc* = 0.85 and *Tc* = 0.5 were taken respectively as the thresholds to indicate high and medium similarities between query molecules and drugs. Finally, the structural similarities between comparatives were determined by the intersection of similarity results by comparing the two results and adopting the lower level of classification as the final similarity outcome for the two conflicting results. Calculation of fingerprints and chemical similarity was performed using CDK Fingerprints and Similarity Search node of Knime (version 3.7.2), respectively [35].

### Functionalities - mechanism exploration of TCM herbs

**Fig. 3:**
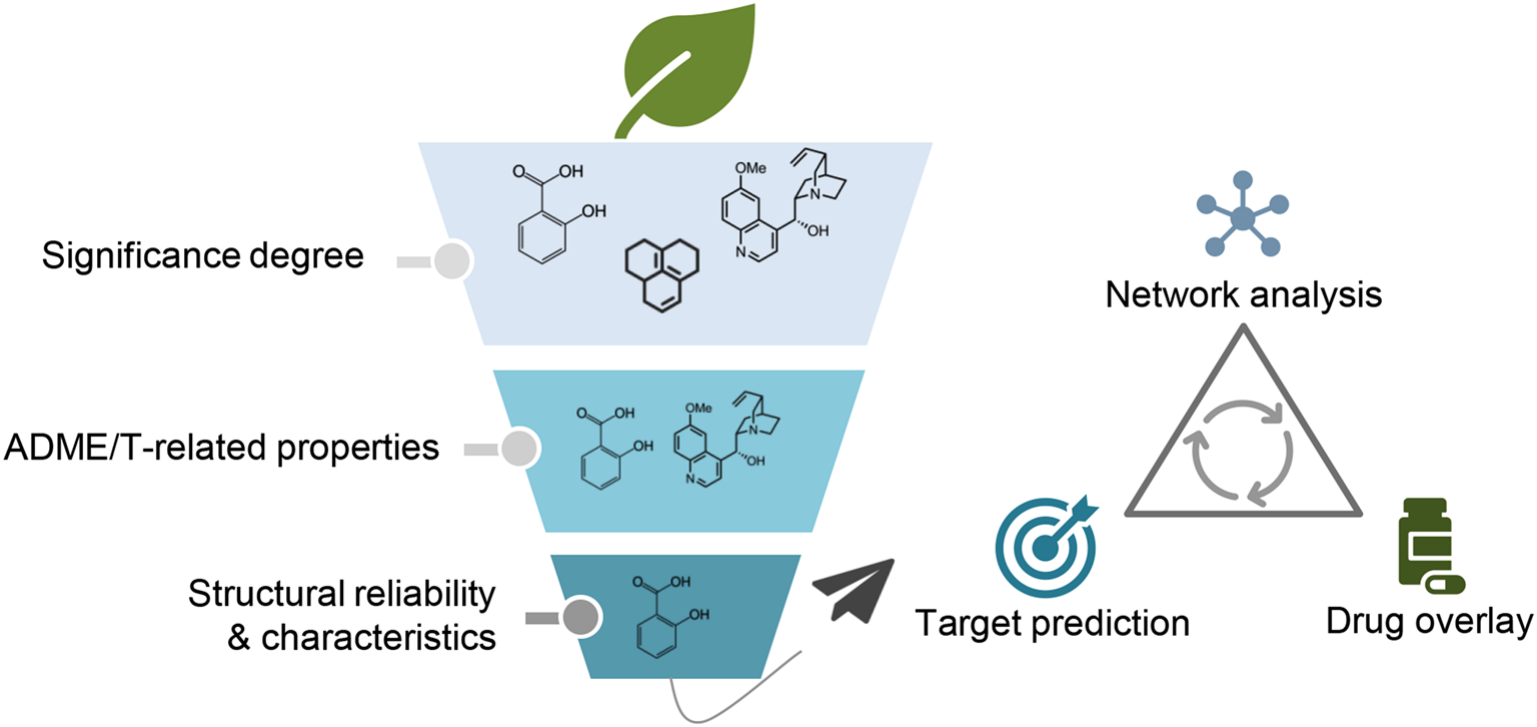
TCM simplification process of TCMSID

To achieve Mechanism exploration of TCM herbs, TCMSID provided TCM simplification for clarifying mechanisms, including two key steps of key ingredients filtering and target identification (Fig. 3). The key ingredients, as the fundamental material basis of TCM, refer to several ingredients that are available to replace a TCM to exert effective pharmacological activity to a certain extent. Key ingredients should have the characteristics of high activity and content. In addition, favorable pharmacokinetic and physicochemical properties should be exhibited to exert potential biological effects. Moreover, given the significant role of molecular structure in pharmacological activity, the structural characteristics and reliability of herbal ingredients should be considered as well. TCMSID provided integrative information for each herbal ingredient, including significance degree, ADME/T and physicochemical properties, structural reliability, structural characteristics, etc. The key ingredients can be filtered in a custom way by setting the threshold range of the above information, according to details of parameters and filtering criteria provided by TCMSID.

Reliable target proteins are the core of mechanism research to promote the modernization of TCM herbs. In recent years, *in-silico* target prediction methods have been regarded as an effective alternative to experimental target identification methods due to its convenience and less time-consuming properties. However, a single target prediction method is more likely leading to inaccurate offset results. It is more beneficial to combine these target prediction methods to take different theoretical foundations into account.

To explore the mechanisms of TCM herbs, the reliable targets of key ingredients can be obtained and aggregated by carrying out multi-tool target prediction. According to the occurrence frequency parameter and detailed target information provided by TCMSID, the potential targets of TCM herbs to exert pharmacological effects can be screened out as well. In addition, TCMSID provides ingredient-related drug information, such as the therapeutic effects and known targets of the drugs being connected, to bridge the gap between TCM herbs and modern drugs through chemical similarity calculation. Finally, the mechanism of action of herbal ingredients can be inferred according to the multilevel herb-ingredient-target-drug network constructed on the network visualization interface.

## Utility and discussion

### User interface

To facilitate the use of TCMSID and make it more convenient and faster, we developed a user-friendly interface for the database. The whole database is deployed on the instance of Elastic Compute Service (ECS) of Alibaba cloud. Considering that the database needs to meet multi-user data access and long transaction, the relational database MySQL from Alibaba cloud was used as the database backend. Also, considering that the database operation needs to be integrated with the cheminformatics computing environment, we use Python as the main coding language for architecture development, since Python development environment provides mature data processing and modeling ecosystems. On this basis, we used the most popular Python-based web framework, Django, combined with HTML5, CSS and JavaScript language to develop the front-end visual interface.

The whole user interface of TCMSID consists of modules of ‘Home’, ‘Browse’, ‘Search’, ‘Help’ and ‘Contact’. In the ‘browse’ module, we show the specific categories of herbs and the structural classification of compounds. The ‘browse’ module is also the main entrance to the database. By expanding the categories of herbs, we can find specific TCM herb and its detailed information. By clicking on the name of the herb, users can get to the main interface of herbal ingredients. On that page, the specific information of herb and the table of ingredients will be displayed. Ingredient tables support some modern operations, such as retrieval, filtering and comparison, which can be realized just by clicking mouse.

In the table, researchers can carry out the first level of TCM simplification. The active ingredients can be simplified and filtered by setting the basic physicochemical properties, structural reliability and ADME/T properties. By clicking the selected ingredient, the detailed information including the identification, basic physicochemical properties, ADME/T results and target prediction results will be displayed on a new page. On this page, we also provide specific information and statistics of targets. Clicking on each target can lead to more detailed information. Here, researchers can simplify the TCM prescription at the second level. By adding a high reliable predicted target and the ingredient itself into the basket which is always affixed at the right bottom of the page, the platform will automatically calculate and generate the network analysis diagram of herb-component-target-drug relationships. Of course, in the ‘browse’ module, the users can directly expand and view the subcategories and ingredients according to the structure category. Users can also get information about ingredients by clicking on the names.

In the ‘search’ module, researchers can carry out general retrieval of ingredients and TCMs according to keywords, and the input supports various types of keywords. In the search result list, users can click on items to display their information. In the ‘Help’ module, we have organized some tutorials on how to use this platform. These tutorials vividly show how to use the above functionalities and customized different analysis pipelines in the form of video. In addition, in the videos, we also showed how to download data and save the results. However, the full database is not publicly downloadable due to server load considerations and website functional design.

### Case study

The TCM formula Erzhi Pill (EZP), composed of *Fructus Ligustri Lucidi* and *Herba Ecliptae*, is one of the frequently used classic prescriptions in China with various pharmaceutical functions such as liver protection and anti-tumor effect [36–39]. However, the mechanism of pharmacological activity exerted by EZP is still unclear, which impeded its application and development. Therefore, implementing TCM simplification to determine the key ingredients and potential targets of EZP, thus further reveal its material basis and mechanism of action are currently urgent problems. Herein, TCM simplification of formula EZP was conducted using TCMSID to illustrate the usage and the validity of the database.

To acquire the key ingredients, a total number of 414 ingredients of EZP were firstly retrieved from TCMSID. With the filtering criteria of Significance degree < 2, Structural reliability < 4, Druglikeness = 1 and Caco-2 > − 5.5, six key ingredients were screened out as the representative of EZP, including oleanolic acid, ursolic acid, salidroside, nuezhenide, specnuezhenide and wedelolactone. After literature research, we found that these six ingredients are key active ingredients of TCM herb *Fructus Ligustri Lucidi* and *Herba Ecliptae* [37–40]. Therefore, we have enough reason to believe that the key ingredients identification process of EZP is accurate and reliable.

**Fig. 4:**
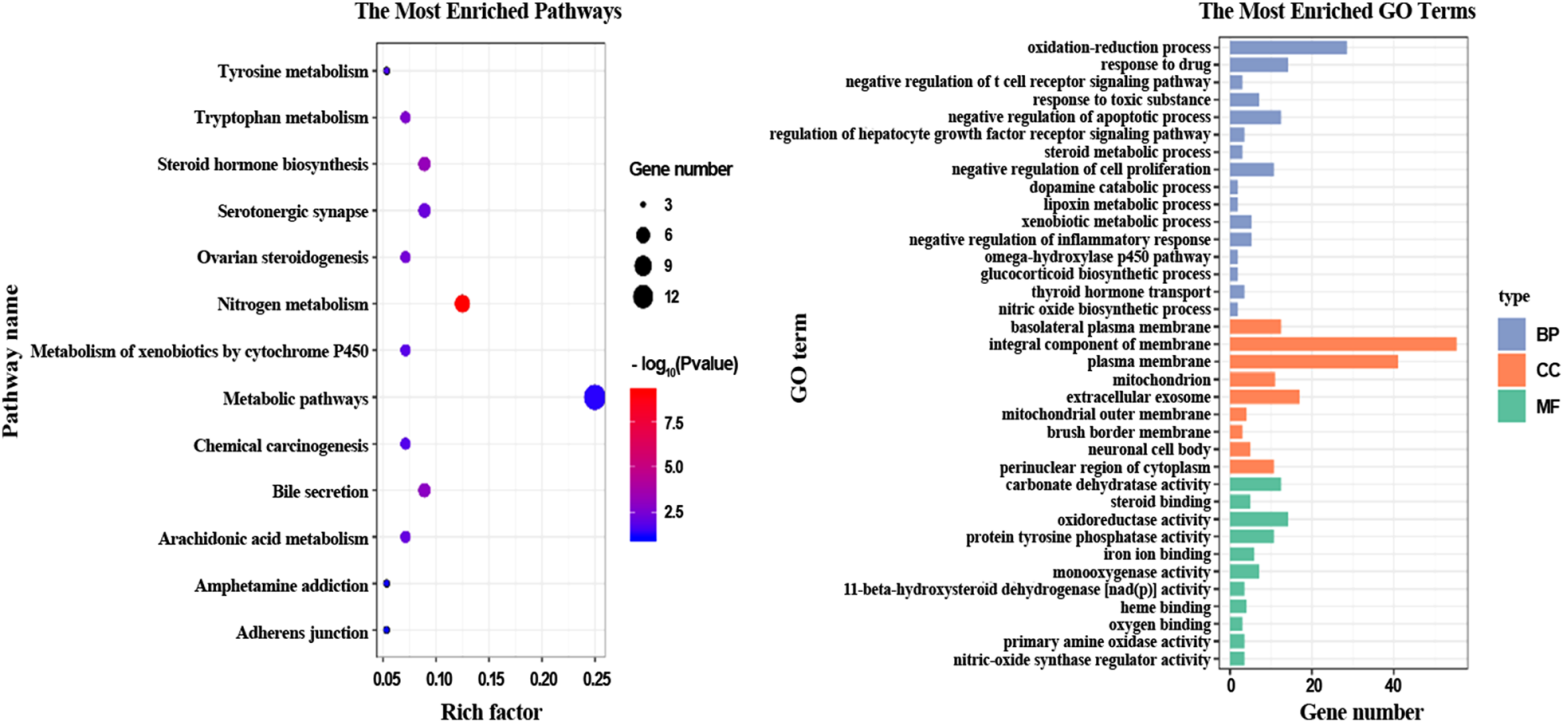
The top enriched GO terms and KEGG pathways for the potential target proteins of formula EZP through functional enrichment analysis by DAVID 6.8. (BP, MF, and CC represent Biological Process, Molecular Function, and Cellular Component groups of GO, respectively)

**Fig. 5:**
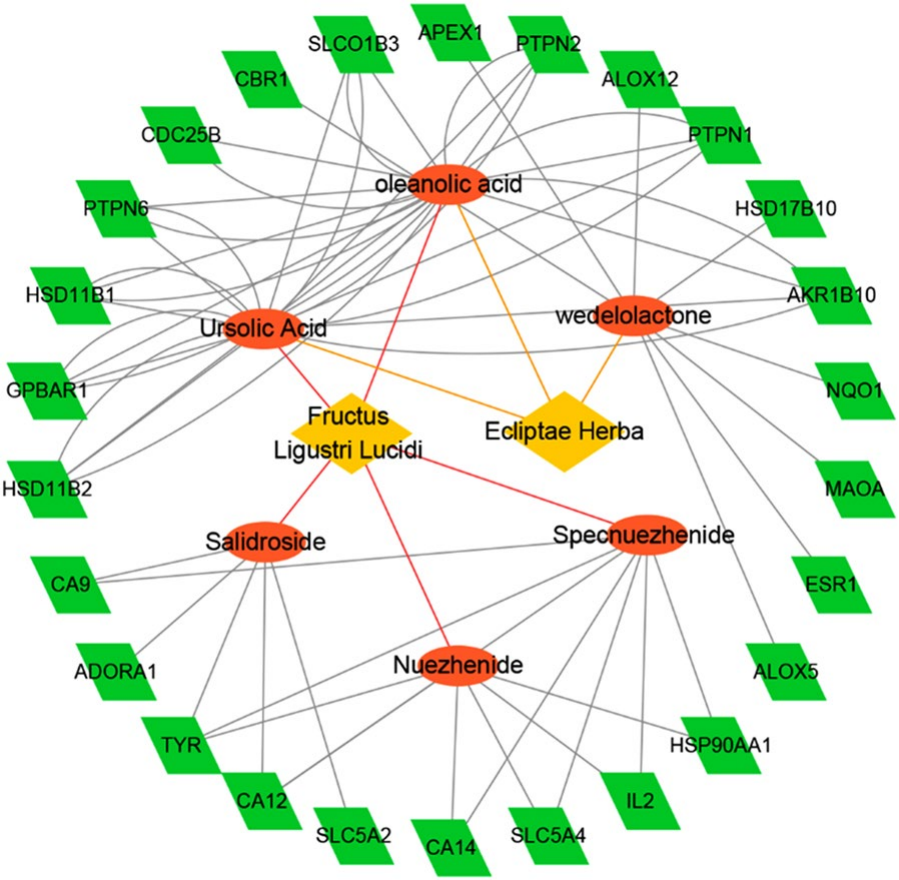
EZP—key-ingredient—target network (generated by Cytoscape 3.5.1)

To obtain the reliable targets of EZP for mechanism analysis, occurrence frequency = 3 was taken as the identification threshold, which means that only target proteins predicted by at least 3 target prediction tools can be identified. Finally, 56 target proteins that met the condition were picked out as the potential targets. To verify the effectiveness of these target proteins, the enrichment analysis was further implemented by Diversity Visualization Integrated Database (DAVID, version 6.8) [41]. As is shown in Fig. 4, we found that these target proteins were closely related to the liver protection and anti-tumor pharmacological activities of EZP through the top enriched KEGG pathways and GO terms[42], such as hsa00910 Nitrogen metabolism, hsa00140 Steroid hormone biosynthesis, hsa00380 Tryptophan metabolism, GO: 0050860 negative regulation of T cell receptor signaling pathway and GO: 0008285 negative regulation of cell proliferation. According to the target-related information provided by TCMSID, 25 target proteins were further identified as the key targets of pharmacological activities exerted by EZP by reference to target classification and reduction of targets and checking irrelevant function. These key targets were closely related to the genes responsible for occurrence and development of liver diseases and tumors, such as CBR1, PTPN1, NQO1, ALOX12, HSD11B2 and HSP90AA1 (Fig. 5, generated by Cytoscape 3.5.1) [43–49]. In a word, we believed that the TCM simplification and mechanism analysis of EZP can be conveniently achieved through TCMSID.

From the selection of herbs in the formulation, to the screening of key ingredients, to the identification of target prediction targets, users can customize the process in each of these steps according to the parameters they choose. As a result, the constructed EZP—key-ingredient—target visualization network is concise but reliable, which can further clarify the mechanism of action of TCM herbs or formulas. For the prediction results from each module, TCMSID provides download options to these analysis results in multiple file formats as well as external links for more detailed information.

Despite of the convenient function of filtering key ingredients of TCM herb or formulas and predicting target proteins potentially connect to these ingredients, the platform still has some limitations require further discussion and improvement. The role of “Jun-Chen-Zuo-Shi” (also known as “sovereign-minister-assistant-courier”) classifies prescription TCM herbs by the importance or the role each of them are playing in. The TCM formula data, such as formula component and the Jun-Chen-Zuo-Shi labeling, is of great importance when users search key ingredients herb by herb. By knowing the formula content, especially the herb importance labeling, users can choose to query herbs based their own decision referring to the role of each herb. Data quality is essential for building prediction models. Better computer-aided quality control methods should be further developed for building a database with higher quality data and more accurate prediction functions.

## Conclusion

To accelerate the progress of TCM’s modernization and standardization, we have presented a Traditional Chinese Medicine Simplified Integrated Database (TCMSID). It is a high storage, high-quality and standardized database, with comprehensive information of ingredients, which largely compensates for the shortcomings of the existing databases. Most importantly, it is not only a data repository just available for information queries, but also a unique mechanism analysis platform for TCM simplification. In short, TCMSID provides data sources and novel research mentality for TCM mechanism research and innovative drug discovery, and it will continue to be developed and updated in the future to promote the modernization and internationalization of TCM.

## Data Availability

TCMSID is freely available at https://tcm.scbdd.com.

## Abbreviations

TCM: Traditional Chinese Medicine
CNKI: China National Knowledge Infrastructure
PPB: Plasma protein binding
BBB: Blood-brain barrier
CL: Clearance
HHT: Human hepatotoxicity
DL: drug-likeness
MW: molecular weight
ECS: Elastic compute service
EZP: Erzhi Pill
